# MOTIVATING PATIENTS WITH PARKINSON’S DISEASE AND THEIR FAMILIES TO DISCUSS PREPARATION FOR END-OF-LIFE CARE

**DOI:** 10.1093/geroni/igac059.3080

**Published:** 2022-12-20

**Authors:** Jiayun Xu, Susan Hickman, Jessica Huber, Rebecca Sudore, Cleveland Shields, Dilhara Moonesinghe, Aubrey Parr, Taylor Kong

**Affiliations:** Purdue University, West Lafayette, Indiana, United States; Indiana University School of Nursing, Indianapolis, Indiana, United States; Purdue University, West Lafayette, Indiana, United States; University of California San Francisco, San Francisco, California, United States; Purdue University, West Lafayette, Indiana, United States; Purdue University, West Lafayette, Indiana, United States; Purdue University, West Lafayette, Indiana, United States; Purdue University, West Lafayette, Indiana, United States

## Abstract

Parkinson’s disease is the second most common neurological illness among older adults. Although most patients with Parkinson’s face communication challenges in advanced illness, few talk about care preferences and are unprepared for worsening illness as a result. Thus, the purpose of the study was to explore the experience of making end-of-life decisions or the preparation of making end-of-life decisions among patients with Parkinson’s, their family caregivers, and clinicians. The long-term goal of this study is to develop a medical decision-making resource for patients and families to better prepare for the end of life. We conducted one-hour semi-structured interviews with nine patients, 12 caregivers, and 12 clinicians to explore perceptions of whether end-of-life discussions may be difficult, the preferred timing of end-of-life information, and how to best motivate patients and their families to prepare. Descriptive thematic qualitative analysis methods were used to analyze the transcribed interviews. Participants were on average 59.7±15.3 years of age, 69.7% female, and 69.7% married. The predominant reasons for delaying end-of-life discussions were the unpredictability of Parkinson’s disease, limited illness and prognostic awareness, and clinicians not initiating the discussion earlier in the disease course. There was a mismatch in preferences for timing of end-of-life information, with patients and family wanting end-of-life information presented earlier in the illness than clinicians. All participants desired a resource to help patients and caregivers cope emotionally, to guide future decisions, and to help prompt conversations. Findings indicate that despite provider concerns, patients and their families want early information about end-of-life issues.

